# Deterioration of eggshell quality in laying hens experimentally infected with H9N2 avian influenza virus

**DOI:** 10.1186/s13567-016-0322-4

**Published:** 2016-02-25

**Authors:** Xuefeng Qi, Dan Tan, Chengqi Wu, Chao Tang, Tao Li, Xueying Han, Jing Wang, Caihong Liu, Ruiqiao Li, Jingyu Wang

**Affiliations:** College of Veterinary Medicine of Northwest A&F University, Yangling, 712100 Shaanxi China

## Abstract

This study aimed to determine the mechanism by which H9N2 avian influenza virus (AIV) affects eggshell quality. Thirty-week-old specific pathogen free egg-laying hens were inoculated with the chicken-origin H9N2 AIV strain (A/Chicken/shaanxi/01/2011) or with inoculating media without virus by combined intraocular and intranasal routes. The time course for the appearance of viral antigen and tissue lesions in the oviduct was coincident with the adverse changes in egg production in the infected hens. The viral loads of AIV have a close correlation with the changes in the uterus CaBP-D28k mRNA expression as well as the Ca concentrations in the eggshells in the infected hens from 1 to 7 days post inoculation (dpi). Ultrastructural examination of eggshells showed significantly decreased shell thickness in the infected hens from 1 to 5 dpi (*P* < 0.05). Furthermore, obvious changes in the structure of the external shell surface and shell membrane were detected in the infected hens from 1 to 5 dpi as compared with the control hens. In conclusion, this study confirmed that H9N2 AIV strain (A/Chicken/shaanxi/01/2011) infection is associated with severe lesions of the uterus and abnormal expression of CaBP-D28k mRNA in the uteri of the infected hens. The change of CaBP-D28k mRNA expression may contribute to the deterioration of the eggshell quality of the laying hens infected with AIV. It is noteworthy that the pathogenicity of H9N2 AIV strains may vary depending on the virus strain and host preference.

## Introduction

H9N2 subtype avian influenza virus (AIV) has been widespread in domestic poultry in Asian countries since the mid-1990s with AIV infections causing mortality ranging from 5 to 30%. H9N2 subtype viruses are classified as low-pathogenicity avian influenza (LPAI) both by molecular characterization and by pathotyping [1]. Chickens infected with H9N2 AIV have mild to severe respiratory signs which include edema of the head and face [2]. It is known that apoptosis and hyper induction of inflammatory cytokines caused by AIV in the respiratory and digestive tracts are major causes of respiratory and digestive failure [3]. In contrast to the extensive work performed in mammalian organisms following influenza virus infection, much remains to be determined about the underlying mechanism of viral infection in the oviducts of egg-laying hens. Our previous study identified that the oviduct is one of the potential targets for the H9N2 subtype AIV virus [4]. These findings showed distinct expression patterns of inflammatory cytokines and chemokines amongst segments of the H9N2 AIV infected oviduct. Although several other studies reported that H9N2 subtype AIV infection in the hen oviduct causes disorders of eggshell formation and reductions in egg production [5, 6], the mechanism underlying these outcomes are not well understood.

The eggshell plays an important role in the resistance of eggs to physical and microbial invasion. Moreover, the eggshell must permit the exchange of gas and water and serve as a source of calcium for the growing embryo. The eggshell is structurally composed of multiple layers, including the external non mineralized cuticle [7], intermediate calcium carbonate crystal layer, and the interior cysteine-rich proteinaceous shell membrane [8]. The eggshell membrane and matrix are formed in the isthmus and uterus of the hen oviduct, respectively. The organic and mineral precursors required for eggshell mineralization are secreted daily by the uterus over a period of approximately 20h into a cell-free medium (uterine fluid), which bathes the egg during the three phases of shell mineralization (initiation, growth and arrest). This fluid contains all the elements (mineral and organic) necessary for shell formation [9]. In the uterine fluid, the organic matrix interacts with minerals and is believed to play a key role in establishing the texture of the shell and its resulting mechanical properties, as observed with other biominerals [10]. Calcium ion for shell formation is secreted from the tubular gland cells of the uterus, and calbindin (CaBP)-D28k plays a primary role in Ca^2+^ transportation [11]. In avian species, high concentrations of CaBP that have been found in the tissues that are characterized by their massive transport of Ca^2+^, such as in the intestine and eggshell gland [12]. In both tissues, CaBP and Ca^2+^ transport are closely correlated [13]. Calbindin is present in the intestine of hens before the onset of reproduction, and its levels increase at the onset of egg production to accommodate the high Ca^2+^ demands for eggshell calcification [14]. In the eggshell gland, calbindin appears during the formation of the first eggshell at the onset of egg production and disappears within 3 days of its cessation [14]. The concentration of calbindin in the eggshell gland is proportional to the rate of shell Ca^2+^ deposition [11, 15]. Whereas a considerable number of studies have addressed the ways in which eggshell characteristics are influenced by factors such as genetics, the time the eggs spend in the uterus, female characteristics, and the diet of the female [16–19], the role of calbindin in determining eggshell quality during infection has received limited attention.

The goal of this study was to elucidate how H9N2 AIV infection in the oviduct of egg-laying hens affects eggshell quality. We examined the effects of chicken-origin H9N2 AIV strain (A/Chicken/shaanxi/01/2011) infection on the *CaBP*-*D28k* gene expression in the uterus and on the calcium contents in the eggshell, as well as on the eggshell ultrastructure of laying hens at different time points post inoculation.

## Materials and methods

### Virus

The virus strain used in this study, H9N2 subtype AIV strain (A/Chicken/shaanxi/01/2011), was isolated from diseased chicken in Shaanxi, China and propagated in 10-day-old embryonated chicken eggs (ECE) at 35 °C for 72 h [20]. All experiments with live viruses were carried out in a biosafety level 3 conditions with investigators wearing appropriate protective equipment and compiling with general biosafety standard for microbiological and biomedical laboratories of Ministry of Health of the People’s Republic of China (WS 233-2002).

### Experimental animals and samples

The study was conducted with 30-week-old specific pathogen free (SPF) White Leghorn egg-laying hens (Green Biological Engineering Co., Yangling, China). All of the hens were housed in isolation units in a biosecure animal building and provided with water and a commercial chicken feed ad libitum (Quality Feed Ltd.). One hundred hens were randomly divided into two groups. The first group of hens (45) was used as uninfected controls. Hens in the second group (55) were inoculated through the combined intraocular and intranasal routes at the age of 30 weeks with a total dose of 10^6^ median embryo infective doses (EID_50_) of the H9N2 AIV strain (A/Chicken/shaanxi/01/2011). After inoculation, the challenged hens were monitored for clinical signs of disease and egg production. At 0, 1, 2, 3, 5, and 7 days post inoculation (dpi), five hens were selected at random from each group, euthanized. The oviducts along with the five parts (infundibulum, magnum, isthmus, uterus and vagina) from controls and experimental groups were collected immediately and placed on ice. Moreover, the uterine tissues with eggs, as well as egg samples, were collected and processed for further analysis. Experimental procedures were undertaken in accordance with the Animal Ethics Monitoring Committee and Animal Welfare Committee of Shaanxi Province, China.

### RNA extraction

Total RNA was extracted from tissue samples (0.1 g) for the indicated days with TRIzol Reagent (Invitrogen, Carlsbad, CA, USA) according to the manufacturer’s instructions. The obtained RNA was quantified with an Agilent 2100 Bioanalyzer using an RNA 6000 Nano Assay kit (Agilent Technologies) plus RNA 6000 ladder marker (Ambion). The purified total RNA from each sample was treated with DNase I to remove potential DNA contamination and was stored at −20 °C for further analyses.

### Quantitative real-time PCR assay for the detection of viral loads

The viral loads of H9N2 AIV in tested sample were quantified by SYBR green-based quantitative real-time PCR assay as described previously [4]. Briefly, the primers targeted to the M gene of H9N2 AIV, forward: 5′-TTCTAACCGAGGTCGAAAC-3′(47 ~ 65), reverse: 5′-AAGCGTCTACGCTGCA GTCC-3′(275 ~ 256) were used for PCR analysis. Viral RNA was reverse transcribed into cDNA for real-time PCR analyses, and the cDNA was further subcloned into the PMD™19-T vector and then transformed into DH5a *E. coli.* The recombinant plasmid was identified by *Eco*RI and *Sal*I digestions. Serial tenfold dilutions of positive recombined plasmid were applied as a positive quantitative template to establish the standard curve from 10^1^ to 10^7^ copies/μL. All amplifications were performed in triplicate. The obtained cycle threshold (Ct) values were plotted against the amount of RNA copy number to the standard curve.

### Histopathology and immunofluorescence examination

Tissues were placed into 10% neutral buffered formalin and fixed for 3 days before being processed for hematoxylin and eosin (H&E) and immunofluorescence (IF) staining for the detection of tissue lesions and AIV antigen, respectively. Procedures used to perform the H&E and IF followed methods previously described [4, 21].

### Real-time RT-PCR analysis for the expression of CaBP-D28k mRNA

Each RNA samples was reverse transcribed with EasyScript First-Strand cDNA Synthesis SuperMix (TransGen biotech, China) and the obtained cDNA stored at −20 °C until required. RT-PCR reactions were performed with equal amounts of cDNA samples and the amplification was carried out using a thermal cycler (Bio-Rad, America). CaBP-D28k primers were designed from a Genbank-registered chicken CaBP-D28k sequence (NM205513). The sense primer (5′-TTGGCACTGAAATCCCACTGA-3′) and antisense primer (5′-CATGCCAAGACCAAGGCTGA-3′) synthesized by the TaKaRa corporation (China), were employed to identify a 116-bp fragment of chicken CaBP-D28k. Beta-actin (β-actin), identified as a stably expressed reference gene, was used as the innate control in this study. The Beta-actin sense primer (5′-AGACATCAGGGTGTGATGGTT-3′) and antisense primer (5′- TGGTGACAATGCCGTGTTCAAT-3′) [22], synthesized (TaKaRa Co., China) and amplified a 118-bp fragment of chicken β-actin. Real-time quantitative PCR and data analysis were carried out using a LightCycler (Roche Diagnostic, Mannheim, Germany) according to the manufacturer’s instructions. The primers for β-actin and CaBP-D28k were the same as those used for standard PCR. Amplification was performed in a total volume of 12 μL, which included LightCycler FastStart DNA Master SYBR Green 1 (Roche Diagnostic). Amplification protocols were performed as previously described [23]. PCR products were electrophoresed on a 1.5% agarose gel, stained with ethidium bromide, and photographed under ultraviolet (UV) illumination.

### Measurement of calcium content in eggshell

Eggshell quality at 0, 1, 2, 3, 5, and 7 dpi was measured within 24 h. At least five eggs were randomly collected from each experimental unit. The eggs were broken and washed, and sample peels of the equatorial region were separated and saved. The membranes of the shell were removed by immersion of samples in a solution of 6% sodium hypochlorite, 4.12% sodium chloride, and 0.15% sodium hydroxide. Afterward, the shells were washed in water and dried at room temperature for 72 h. Then, the calcium content of the eggshell was detected according to the methodology described previously [24].

### Ultrastructural observation for measurement of eggshell quality

Three samples of the shell of each egg (1–2 mm^2^) were used for the analysis of the external shell surface, shell membrane, and cross section of the eggshell. The samples were glued on an aluminum support (stub), metalized with gold, and analyzed with a Shimadzu SS-550 Superscan scanning electron microscope (Shimadzu Corporation, EVISA, Kyoto, Japan). For the analysis of the cross-section of the shell, the total thickness was measured in three equidistant locations around the equator of the egg [18].

### Statistical analysis

All described experiments were performed a minimum of five times, and the data were calculated as the mean ± standard error of mean (SEM). The effects of AIV infection on all dependent measures were analyzed with two-way analysis of variance (ANOVA). Significant interactions were further analyzed using the Tukey method for pairwise multiple comparisons. A *P* value of less than 0.05 was regarded as statistically significant. The data shown in some figures (e.g., photographs of scanning electron microscope analysis) are from a representative experiment, which was quantitatively replicated in at least five independent experiments.

## Results

### Viral loads in oviduct

**Figure 1:**
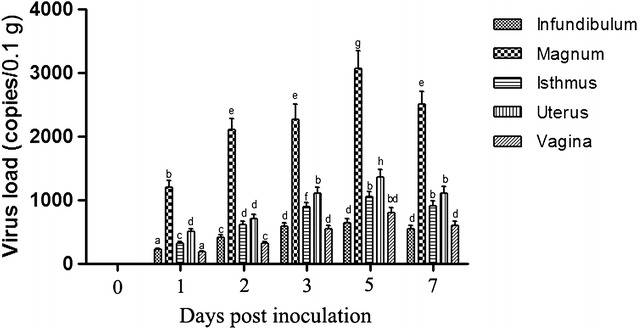
**Viral loads in the oviducts of H9N2 subtype avian influenza virus (AIV) infected laying hens.** Kinetics of virus load of H9N2 subtype AIV in the oviducts of laying hens infected with H9N2 subtype AIV strain using quantitative real-time PCR. Each time point represents the mean viral genome copies per 0.1 g of tissues ±SEM obtained from five hens.

Absolute quantitative detection of virus load by real time quantitative PCR demonstrated that different AIV DNA loads were observed in five parts (infundibulum, magnum, isthmus, uterus and vagina) of the oviducts in inoculated hens at 1 dpi. Thereafter, the viral loads in the oviduct increased by various rates until 5 dpi and followed by a decrease in virus load at 7 dpi (Figure 1). It is important to note that the viral loads of AIV measured in magnum and uterus was significantly higher as compared with that measured in infundibulum, isthmus, and vagina tissues at 1, 3, and 5 dpi.

### Clinical signs, histopathology and viral distribution in oviduct

**Figure 2:**
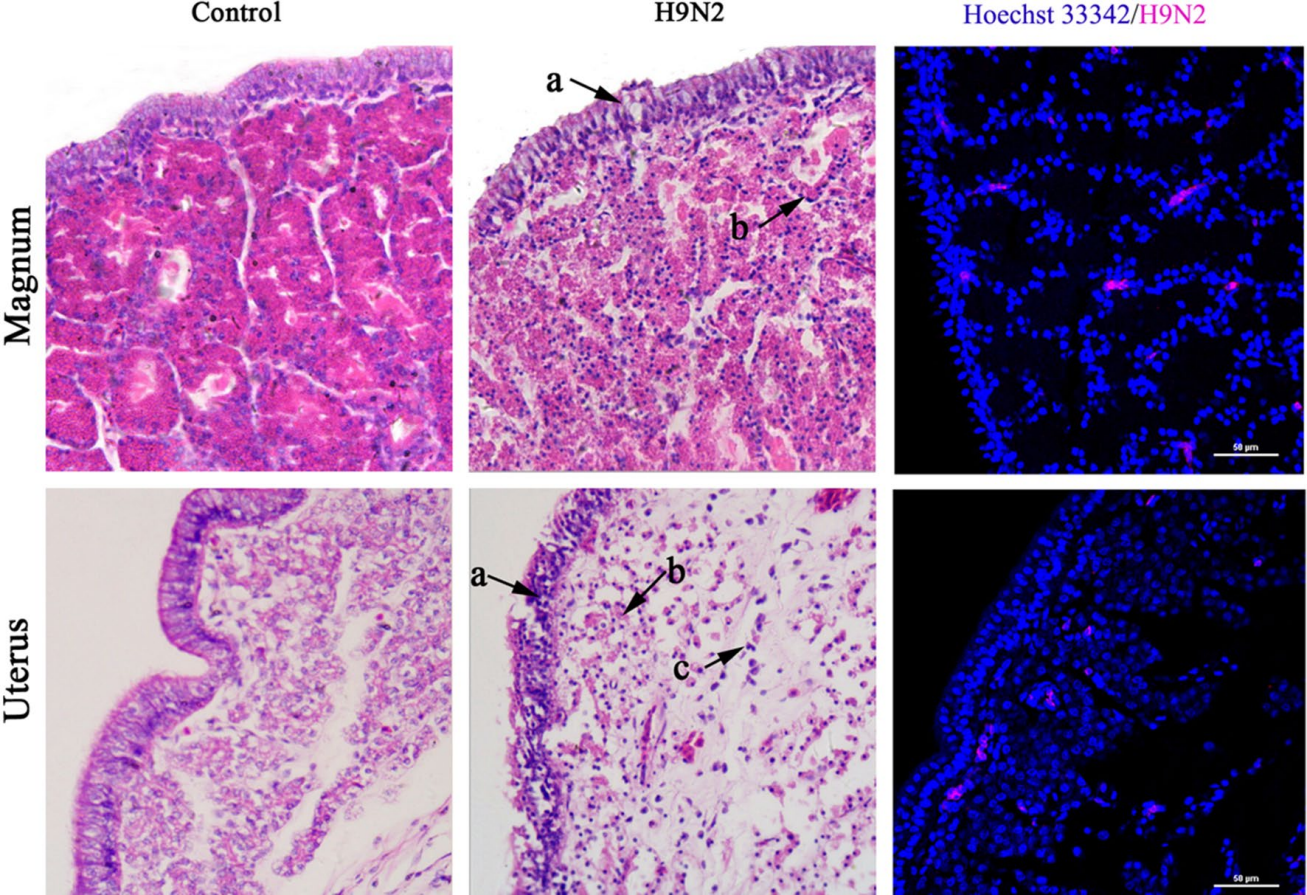
**Histopathologic changes and the location of virus antigen in the magnum and uterus of hens infected with H9N2 subtype AIV.** Histopathologic changes of the magnum and uterus in control and AIV-infected groups on day 5 post inoculation was observed by hematoxylin and eosin (H&E) staining (400×). (a) degeneration and necrocytosis of mucosal epithelial cell layer; (b) glands edema or dissolved; (c) infiltration of lymphocytes. Nuclei of the tissue sections from control and infected hens were stained with Hoechst 33342: red colour fluorescence indicates the positive staining of virus NP proteins. Scale bar = 50 µm.

Clinical signs of the infected chickens were observed from the third day of inoculation. Infected chickens were depressed and showed signs of respiratory symptoms. They were consuming less food and water and producing yellow–white, viscous loose stools. A gradual drop in egg production appeared immediately following H9N2 AIV inoculation. The results obtained in the current study showed that egg production of H9N2 AIV-inoculated hens dropped to approximately 60% by 7 dpi, whereas control chickens maintained approximately 95% egg production. Furthermore, infected chickens were characterized by laying deformed or soft-shelled eggs which on necropsy were discovered to have follicles that were hemorrhagic, congestive or dissolved with some oviducts showing signs of edema and seeping out of white jelly like material. The control hens did not manifest any signs of disease throughout the infection, nor was egg production affected. Histopathologic examination in the present study showed that the mucosal epithelium of the magnum and uterus in the control hens was lined by a ciliated pseudostratified epithelium, and the lamina propria was filled with tubular glands (Figure 2). However, severe lesions in the magnum as well as uteri of hens infected with AIV were detected throughout the infection. As shown in Figure 2, the mucosal epithelial layer of both the magnum and uterus showing signs of degeneration, necrocytosis or fallen off. Furthermore, degeneration, necrocytosis or fallen off of some glandular epithelial cells in lamia propria of the magnum (arrow) were observed at 5 dpi. In the uterus of 5 dpi, edema of the tubular glands and a large infiltration of lymphocytes in the lamina propria (arrow) were observed. In addition, IF detection revealed that H9N2 AIV antigen was found most abundantly in the glandular epithelium of the infected magnum and uterus (Figure 2).

### Change of CaBP-D28K gene expression in uterus

**Figure 3:**
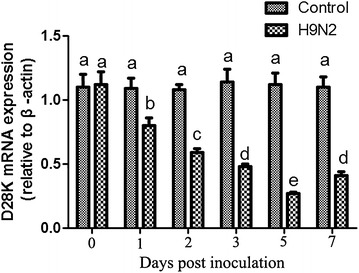
**The expression of CaBP-D28k gene in the uterus of laying hens at different time points post inoculation with AIV.** Analysis of CaBP-D28k mRNA expression in the uterus of laying hens at the indicated days post inoculation with AIV using quantitative RT-PCR. Columns and vertical bars represent the mean ± SEM from five independent experiments. Bars with different superscript letters represent a significant difference (*P* < 0.05).

The quantitative detection of CaBP-D28k mRNA expression levels in the oviducts of control and infected hens showed that significantly higher levels of CaBP-D28k mRNA was detected in the uterus throughout the experiment as compared with other tissues examined, including infundibulum, magnum, isthmus, and vagina, which is very low or undetectable in CaBP-D28k mRNA expression (data not shown). It is important to note that the levels of CaBP-D28k mRNA expression in the uterus was significantly lower in the AIV-infected group than in the control group during 1~7 dpi, particularly at 5 dpi (*P* < 0.05; Figure 3).

### The content of calcium in eggshells

**Figure 4:**
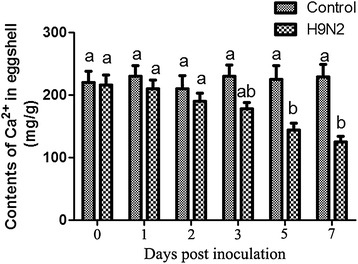
**Analysis of the content of calcium in the eggshell of laying hens at different times post inoculation with AIV.** Analysis of the content of calcium in the eggshell of laying hens at the indicated days post inoculation with AIV using flame atomic spectrophotometry. Columns and vertical bars represent the mean ± SEM from five independent experiments. Bars with different superscript letters represent a significant difference (*P* < 0.05).

The change in eggshell calcium content was analyzed by flame atomic spectrophotometry in the present study. As shown in Figure 4, a significant decreased level of calcium content was detected in the eggshells of infected hens during 3~7 dpi (*P* < 0.05), and a slight increase of calcium content was detected at 7 dpi.

### Ultrastructural observation of eggshell

**Figure 5:**
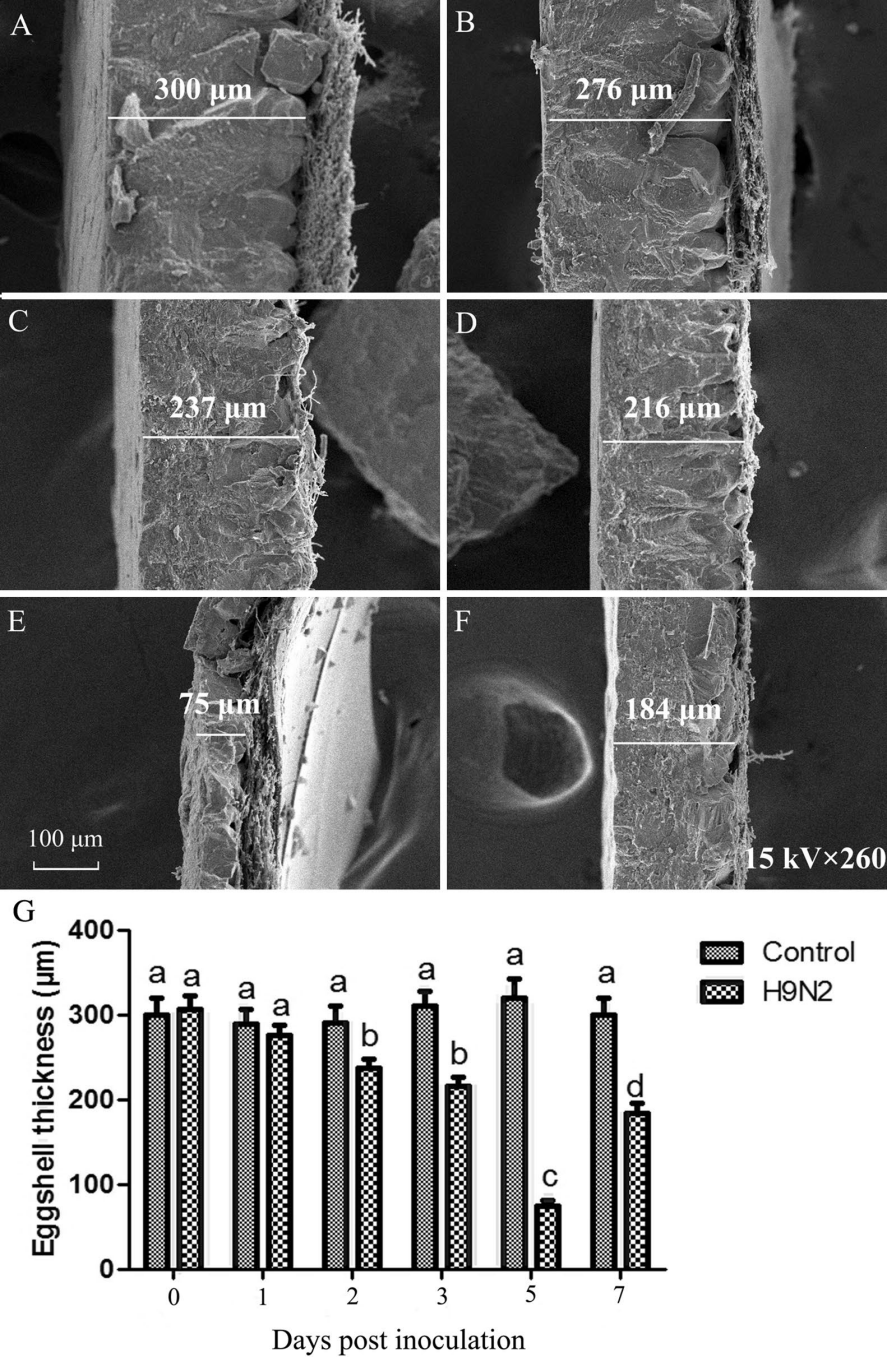
**Scanning electron microscopy of the cross section of the eggshells from laying hens at different times post inoculation with H9N2 AIV.** Representative scanning electron microscopy photograph of the cross section of the eggshell in the control group (**A**) and AIV-infected group at 1~7 dpi (**B**–**F**) as well as analysis of the eggshell thickness (**G**) in laying hens at the indicated days post inoculation with AIV. Columns and vertical bars represent the mean ± SEM from five independent experiments. Bars with different superscript letters represent a significant difference (*P* < 0.05). Scale bar = 100 µm.

**Figure 6:**
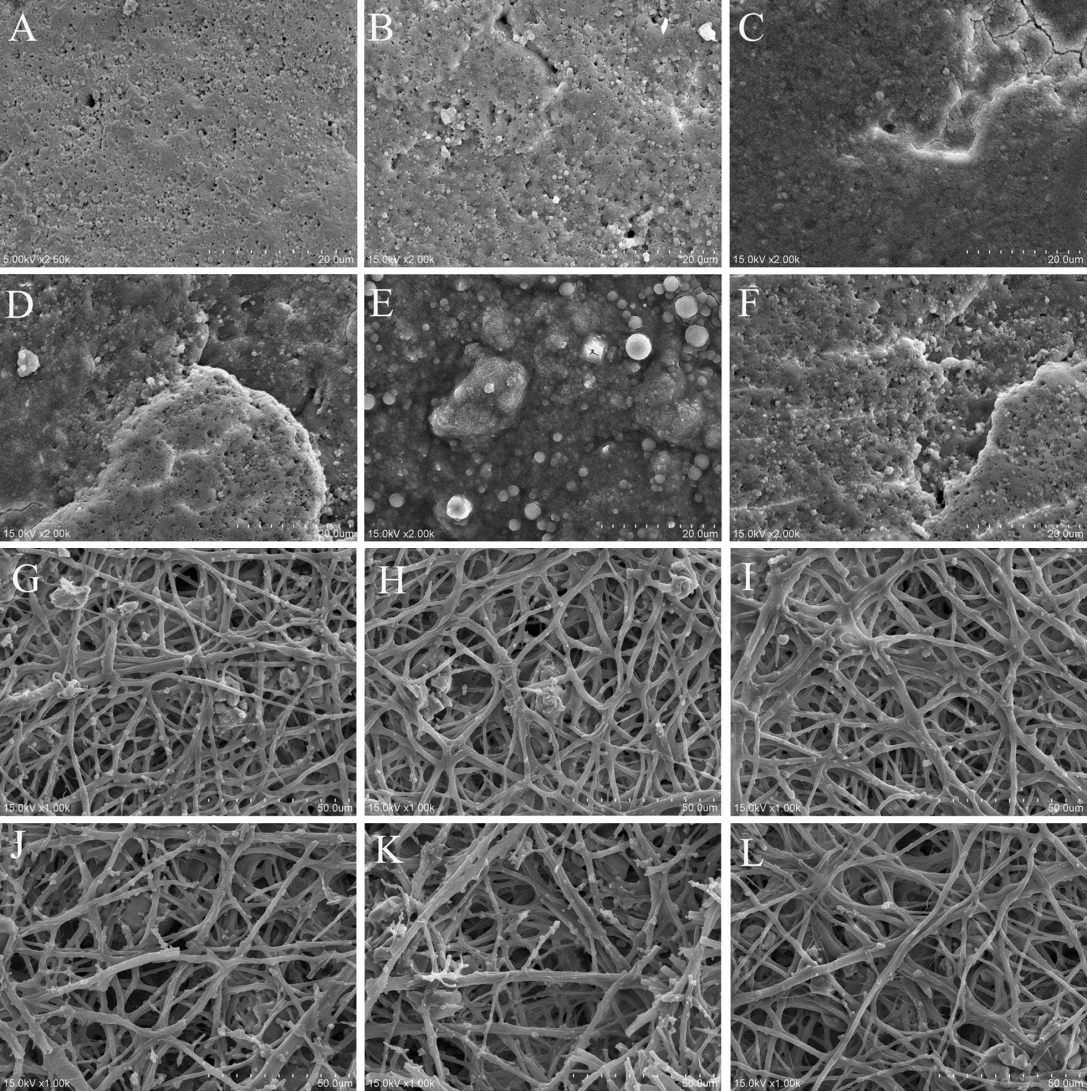
**Scanning electron microscopy of the inner and outer surface of the eggshells of laying hens at different times post inoculation with AIV.** Representative scanning electron microscopy photograph of the outer (**A**–**F**) and inner surface (**G**–**L**) of the eggshells from the control hens (**A**, **G**) and in hens at 1~7 days post inoculation with AIV (**B**–**F**, **H**–**L**).

The quality of eggshell in AIV infected hens was examined by scanning electron microscope. As shown in the Figure 5, eggshell thickness decreased significantly compared with that of the control as early as on day 2 post inoculation (*P* < 0.05), and the thinnest shells was detected on day 5 post inoculation (*P* < 0.05). Although significantly increased shell thickness was observed at 7 dpi, a significant decrease in egg thickness was detected in the hens compared with that of the control group (*P* < 0.05; Figure 5G). Ultrastructural observation on the changes of the normal structure of the external shell surface showed a smooth, clear structure, with a few gas pores scattered on the external surface of the eggshell in control hens (Figure 6A), whereas more pores, crevices along the pores, and embossment were observed on the external surface of the eggshells from hens inoculated with AIV for the period from 1 dpi to the end of the experiment (Figures 6B–F). Particularly, evident embossment as well as more small holes on the shell surface was detected in infected hens at 3~5 dpi (Figures 6D and E). Furthermore, the membrane in control hens showed a compact and well-organized fibrous layer (Figure 6G). However, more fractured fibers, as well as a loose fibrous layer were observed in the shell membrane in infected hens during 2~7 dpi (Figures 6I–L), and more thick fibers with many small granular bumps in the fibrous layer were detected during the period from 3 to 5 dpi (Figures 6J and K).

## Discussion

To our knowledge, this study is the first to report that the viral loads of H9N2 subtype AIV have a close correlation with the changes in the uterus CaBP-D28k mRNA expression, and the abnormal expression of CaBP-D28k mRNA are associated with the declines in egg production and deterioration in eggshell quality in H9N2 AIV-infected laying hens.

In the present study, the kinetics of the viral loads and localization of AIV antigen in the oviduct of laying hens inoculated with H9N2 subtype AIV isolate (A/Chicken/shaanxi/01/2011) was investigated. Our results showed that different AIV loads were detected in the oviduct in infected hens throughout the experiment. AIV preferentially binds to SA α-2,3-galactose (SAα2,3-gal) linked receptors, while human strains bind to sialic acid α-2,6-galactose (SAα2,6-gal) linked receptors [25, 26]. The type and distribution of SA receptors are critical for the replication of AIV. Our previous findings revealed that SAα2,3-gal receptor were dominant in the epithelia and infrequent in the tubular glands of magnum and uterus [27]. In the present study, AIV viral loads in magnum and uterus were significantly higher than those in isthmus and vagina, indicating that the higher viral loads that caused several pathological changes in the tissue were positively correlated with the expression of SA receptors. Furthermore, the oviduct is an independent organ in laying hens which has a certain influence on AIV transmitting through the eggs. As already suggested [28], the virus replication in the oviduct supports the presence of virus in the yolk and albumin of eggs from infected hens. Although avian H9N2 virus in the eggs was not detected in the current study, we can’t rule out the possibility of the presence of H9N2 subtype AIV in eggs from infected hens.

It has been shown that egg-laying hens infected with H9N2 AIV show symptoms of mild bleeding in the respiratory, digestive and reproductive tracts [29–31]. Interestingly, severe lesions in the magnum as well as uteri of hens inoculated with H9N2 AIV strain (A/Chicken/shaanxi/01/2011) were detected in the present study. Variation in the pathogenicity of different strains of H9N2 AIV has been demonstrated in previous studies [32]. Although H9N2 AIV strain (A/Chicken/shaanxi/01/2011) used in this study does not satisfy the criteria for highly pathogenic AIV, our genetic findings suggest that there is an N383D substitution at residue 383 in the RNA polymerase subunit A (PA) protein [20], which may increase the virulence of the virus [33]. Another possible explanation is that the pathogenicity of different strains of H9N2 may simply vary depending on the host preference [34]. Chicken-origin H9N2 AIV strain (A/Chicken/shaanxi/01/2011) used in this study may replicate efficiently in laying hens and cause more severe tissue damage. In addition, in contrast to mild lesions in reproductive tract in laying hens natural infected with H9N2 AIV reported by previous studies [29–31], inoculation routes as well as inoculated viral dose may also cause severe tissue damage in oviduct observed in the current study. In any event, our data suggest that the H9N2 AIV strain (A/Chicken/shaanxi/01/2011) can cause severe pathological changes in the oviduct of laying hens.

The high post challenge AIV-specific positive labeling in the tissues of the oviduct indicated that the infected cells could be impeded from performing their specialized functions, and in this case, reduced egg production could be the result. Not surprisingly, the hens inoculated with H9N2 AIV strain (A/Chicken/shaanxi/01/2011) experienced significant drops in egg production was detected in this study. These findings are in line with previous natural and experimental low-pathogenicity AIV infections of laying hens [27, 35].

Because both the lining and glandular epithelium of the oviduct exposed to the virulent AIV challenge virus were affected, as evidenced by the presence of AIV antigen in this tissue, the expression of genes related to eggshell quality in the uterus may be severely affected in the H9N2 AIV-inoculated hens. CaBP-D28k was reported to be expressed in the tubular gland cells of the uterus and to play a vital role in the transportation of Ca^2+^ for eggshell formation [11, 36]. Furthermore, besides the suggested role of calbindins in Ca^2+^ transport, they may also be involved in protecting the cells from high concentration of Ca^2+^ or from cellular degradation via apoptosis and also it may act as a buffer [37]. However, the role of calbindin in determining eggshell quality under pathogen infection has received limited attention. In the current study, the expression of CaBP-D28k was significantly lower in the AIV group than in the control group during 1~7 dpi, especially at 5 dpi. It is worth pointing out that the changes of AIV loads in the uterus are closely correlated with the changes of uterus CaBP-D28k mRNA expression. Thus, it is suggest that H9N2 AIV strain (A/Chicken/shaanxi/01/2011) infection may affects the expression of CaBP-D28k in the uterus, leading to disruption of the eggshell membrane and eggshell formation. However, further studies are still needed to clarify the role of H9N2 AIV strain (A/Chicken/shaanxi/01/2011) infection in CaBP-D28k expression in other organs, such as kidney, brain, intestine, and eggshell gland. Moreover, our recent study have demonstrated that immune-related genes, including cytotoxic immunoreaction and proinflammatory cytokines showed variation in the egg laying hens infected with H9N2 AIV strain (A/Chicken/shaanxi/01/2011) [4]. Similar results obtained in previous study showed that avian infectious bronchitis virus infection causes disorder of eggshell formation by disturbing gene expression of CaBP-D28k in the uterus via the effects of substances from cytotoxic cells and proinflammatory cytokines [38].

The eggshell is a complex and highly structured calcitic structure. Approximately 94% of eggshell mineral is calcium carbonate, with other inorganic minerals such as magnesium carbonate, calcium phosphate and magnesium phosphate [39]. It is important to note that the kinetics of CaBP-D28K mRNA expression coincided with the change in eggshell calcium content analyzed by flame atomic spectrophotometry in the current study. Because calbindin has a significant role in Ca^2+^ transportation which consequently participates in eggshell calcification, it could be assumed that the decreased calcium contents in eggshell of H9N2 AIV strain (A/Chicken/shaanxi/01/2011) infected hens laying eggs might be attributed to low levels of CaBP-D28K mRNA expression in the infected uterus.

The strength of the eggshell is directly related to its thickness and the structure of the external surface as well as the shell membrane. Increased resistance of eggshells is a desirable feature that has economic importance in a commercial laying sector [40]. Despite the eggshell thickness appears to be influenced by many factors including genetics, egg colour and size, the time the eggs spend in the uterus, female characteristics, and the diet of the females [16, 41–43], several studies have shown that pathogen infection also produced egg abnormalities and eggshell thinning [44, 45]. In this study we provide the first quantitative data on eggshell thickness variation of H9N2 AIV strain (A/Chicken/shaanxi/01/2011) infected hens laying eggs. Our results are consistent with the prediction that eggshell thickness decreases during AIV infection. To our knowledge, the effect of lowly pathogenic AIV infection on eggshell thickness variation has never been examined before. Furthermore, changes of the normal structure of the external shell surface and the shell membrane are associated with reduced shell strength as well as less resistance to microbial invasion. Ultrastructural observation of the eggshell in this study showed deterioration of eggshell quality in AIV infected hens on days 2~7 post inoculation. Decreased calcium contents induced by AIV infection may, in part, contribute to the deterioration in eggshell quality in AIV-infected hens. It is noteworthy that the deterioration in eggshell quality in H9N2 AIV-infected hens may vary according to the virus strain and host preference.

In conclusion, we confirmed that lowly pathogenic H9N2 AIV strain (A/Chicken/shaanxi/01/2011) infection is associated with severe uterine lesions and decreased expression of CaBP-D28k mRNA in the uteri of egg-laying hens. Such disorder in the expression of eggshell components may be responsible for the decline in egg production as well as the deterioration of eggshell quality in hens infected with H9N2 subtype AIV strain (A/Chicken/shaanxi/01/2011). It is worth noting that not all H9N2 subtype AIV isolates can cause severe oviduct damage observed in this study in view of variation in the pathogenicity of different strains of H9N2 AIV. Further studies are required to determine the variation in reproductive tract lesions of laying hens in response to different H9N2 isolates infection and infection routes.

## Abbreviations

AIV: avian influenza virus
SPF: specific pathogen free
CaBP: calcium-binding protein
H&E: hematoxylin and eosin
IF: immunofluorescence
